# Social cognition in mild cognitive impairment and dementia: a systematic review and meta‐analysis

**DOI:** 10.1002/alz70857_099687

**Published:** 2025-12-24

**Authors:** Puyu Shi, Hannah Chapman, Lisa Liu, Fern Rodgers, Jasmine Shaw, Gill Livingston, Katherine P Rankin, Jason D Warren, Andrew Sommerlad

**Affiliations:** ^1^ University College London, London, London, United Kingdom; ^2^ Queen Mary University of London, London, United Kingdom; ^3^ Camden and Islington NHS Foundation Trust, London, London, United Kingdom; ^4^ Memory and Aging Center, University of California San Francisco, San Francisco, CA, USA; ^5^ Dementia Research Centre, UCL Queen Square Institute of Neurology, London, London, United Kingdom

## Abstract

**Background:**

Social cognition is crucial for successful social interactions and maintaining social relationships, and is a core cognitive domain in DSM‐5 diagnostic criteria for dementia. Previous literature has shown that social cognition is more impaired in people with dementia compared to healthy older adults, as well as in people with Mild Cognitive Impairment (MCI) compared to healthy older adults. However, the clinical distinction in social cognition between those with MCI and progressive dementia remains underexplored. Understanding these differences may help clarify the predictive role of social cognition in the progression from MCI to dementia

**Methods:**

We systematically searched databases for studies comparing social cognition between people with MCI and dementia. Separate meta‐analyses were performed to calculate effect sizes for standardised differences in emotion recognition, Theory of mind (ToM), and empathy (cognitive and emotional empathy).

**Results:**

From 2,490 identified studies, 28 cross‐sectional studies involving 2,368 participants (1,273 MCI, 1,145 dementia) met inclusion criteria. People with MCI had better emotion recognition (Cohen's d = 0.69) and ToM (Cohen's d = 0.74) abilities than those with Alzheimer's disease (AD) dementia. Larger differences were observed between people with MCI and frontotemporal dementia (FTD) (emotion recognition Cohen's d = 2.09 and ToM Cohen's d = 1.49). People with AD showed slightly higher emotional empathy compared to those with MCI in all included studies, though this difference was not significant. No significant difference was found for cognitive empathy.

**Conclusion:**

Our findings demonstrate differences in social cognition between MCI and dementia, particularly in emotion recognition and ToM, with more pronounced impairments in people with FTD than those with AD. These findings support that social cognition declines with disease progression and suggest the clinical value of social cognition assessments in distinguishing people at risk of progressing from MCI to dementia. Studies should develop and evaluate targeted interventions for social cognition in people with MCI or early dementia. Longitudinal studies are needed to further clarify the predictive role of social cognition in the progression from MCI to dementia, and to explore how these deficits vary across MCI subtypes and relate to specific dementia outcomes.

